# Whitefly attraction to rosemary (*Rosmarinus officinialis* L.) is associated with volatile composition and quantity

**DOI:** 10.1371/journal.pone.0177483

**Published:** 2017-05-12

**Authors:** Dganit Sadeh, Nadav Nitzan, Alona Shachter, David Chaimovitsh, Nativ Dudai, Murad Ghanim

**Affiliations:** 1 Unit of Aromatic and Medicinal plants, Newe Ya’ar Research Center, ARO, Ramat-Yishay, Israel; 2 Department of Entomology, Volcani Center, ARO, Bet Dagan, Israel; 3 Robert H. Smith Faculty of Agriculture, Food and Environment, Hebrew University of Jerusalem, Rehovot, Israel; Zhejiang University, CHINA

## Abstract

Whitefly (*Bemisia tabaci*) is an important insect pest, causing severe damage to agricultural crops. The pest was recorded in a commercial rosemary (*Rosmarinus officinalis*, Lamiaceae) field, colonizing rosemary variety (var.) '2', but not '11'. A series of field and controlled laboratory choice bioassays confirmed the observed phenomenon. Mature potted plants of the two varieties were randomly organized in a lemon verbena (*Lippia citrodora*) and lemon grass (Cymbopogon spp.) fields. Seven days later var. '2' was significantly more colonized by whiteflies than var. '11'. Under lab conditions, whiteflies were significantly more attracted to var. '2' plantlets than to var. '11' following choice bioassays. Furthermore, cotton plants dipped in an essential oil emulsion of var. '2' had significantly greater colonization than cotton plants dipped in the essential oil emulsion of var. '11'. Similar results were obtained in 'plant-plant', 'plant-no plant' as well as, 'essential oil—essential oil' choice bioassay designs. Analyses of the essential oils of the two varieties identified a set of common and unique volatiles in each variety. Among these volatiles were β-caryophyllene and limonene, two compounds known to be associated with plant-insect interactions. The attraction of *B*. *tabaci* to pure (>95%) β-caryophyllene and limonene using a range of concentrations was examined *in vitro* by choice bioassays. The compounds were attractive to the insect at moderate concentration, but not at the lowest or highest concentrations used, where the insect was not attracted or repelled, respectively. Limonene attracted the insects at rates that were 10-fold lower than β-caryophyllene. The results emphasized the role of host plant volatiles in shaping the structure of *B*. *tabaci* populations in nature and in agricultural systems, and provided insights into the factors that contribute to the development of insect populations with unique characteristics. The results could also serve for future development of bio-pesticides and in breeding programs.

## Introduction

The whitefly *Bemisia tabaci* (Hemiptera: Aleyrodidae; sweet potato or tobacco whitefly) is a cosmopolitan, broad-spectrum phloem-feeding insect that feeds on more than 900 plant hosts. *B*. *tabaci* is considered a species complex, containing over 35 different biotypes or cryptic species [1,2,3,4,5,6,7]. The adaptation of *B*. *tabaci* to a specific host plant is considered biotype-dependent [4]. Within the *B*. *tabaci* species complex, the Middle East-Asia Minor 1 species (MEAM1, previously known as the ‘B biotype’) and the Mediterranean species (MED, previously known as the ‘Q biotype’) are the two most frequently encountered in the last 20 years [5,8,6]. Compared to other cryptic species members, these two whitefly biotypes are more damaging, have greater fitness [9] a longer lifespan [10], more insecticide resistance [11,12], mutual interactions with whitefly transmitted begomoviruses [13,14] and more aggressive mating behaviors [6]. Consequently, they have replaced most of the other indigenous whitefly species in many regions worldwide, including China, southern Africa and Asia [15]. Epidemics of begomoviruses are usually associated with whitefly outbreaks, which act as vectors of viruses from this group [16]. Therefore, limiting the invasive spread of whiteflies is essential in begomoviral disease control worldwide.

Rosemary (*Rosmarinus officinali*s L) is an evergreen plant, a member of the *Lamiaceae* family, which is distributed throughout the Mediterranean, where it is used primarily as a spice herb for culinary purposes. Rosemary essential oil and secondary metabolites are recorded as antimicrobial, anticancerous [17,18], enhancers of cognition [19] and local blood circulations, as well as pain relievers [20] and are used in the cosmetics industry [21,22,23]. Israel produces approximately 500 metric tons of fresh rosemary commercially annually, primarily for export.

In July 2010, during a routine field monitoring, biotype B whiteflies were observed massively colonizing var. '2', but not '11', which are two advanced rosemary germplasm selections developed for industrial production at the Newe Ya’ar, Aromatic and Medicinal Plant Breeding Program of the Agriculture Research Organization (ARO, Volcani Institute). The massive colonization continued also following an aggressive shaking of the leaves. The whiteflies always returned to var. '2', but not to '11'.

Volatiles and chemical compounds emitted from plants are major players in attracting and governing interactions with insects [24,25,26,27]. Essential oils are a rich source of active volatile chemicals [28,29], which can have diverse effects on the insects' respiratory, gastroenterological [30] and neurological systems [27]. The biological properties may be the outcome of single essential oil compounds or of synergism among them [31]. For example, essential oil of *Cunila incana* (Lamiaceae), which is rich in α and β pinene and β- caryophyllene, was toxic to larvae of the cattle tick *Rhipicephalus (Boophilus) microplus* [32]. Soliman [33] found that essential oil of *Artemisia herba-alba* and *A*. *monosperma* were rich in monoterpenes (58.1–57.1%) and had high toxicity to *B*. *tabaci* and *Aphis gossypii* in lab, greenhouse and field trials. Furthermore, Baldin et al. [34] report that the essential oil of *Pelargonium graveolens L’Her* (Geraniaceae) (PG-EO) and its related monoterpenes, geraniol, linalool, and citronellol, significantly reduced the number of *B*. *tabaci* adults on tomato. Deletre et al. [35] reported that geraniol and citronellol posessed repellent, irritant and toxic activities, depending on their applied concentrations, and those activities were independent for both compounds. Hence, could be used in coating nets for repellent activities in nethouses. Tosh et al. [36] studied the confusion effect on whiteflies by supplying headspace with different plant hosts to whiteflies and measuring effects on their feeding behavior and egg lay. The results indicated that no such effects were observed; suggesting that these volatile mixtures are not strong in preventing and controlling whitefly performance.

Mena et al. [37] reported that terpenes like verbenone, bornyl acetate, camphor, and α/β caryophyllene represented more than 24% of the total volatiles in rosemary extracts. In light of this information the present study tested the hypothesis that variations in volatiles associated with varieties '2' and '11' are the cause of the differential attraction of *B*. *tabaci* to them. The objectives of the study were to validate and quantify the whitefly attraction and repellency traits of each rosemary variety in controlled environments, and to elucidate the volatile compounds that are associated with this phenomenon.

## Materials and methods

### Plant material

Plants of varieties (var.) '2' and '11' from the germplasm bank at the Newe Ya'ar Research Center campus of the Agriculture Research Organization were propagated by stem cuttings [38]. The propagation cycles were carried out once the plants reached the height of 5–15 cm. The stem cuttings were propagated in 50 mL flowerpots in the greenhouse, under a 70% light-reducing shade net. The plants were irrigated once a week using a drip irrigation system following a 70% evapotranspiration factor. Fertilization was added to the irrigation water at a 1.5 Kg of pure nitrogen per 1 ha/per day. Plantlets were used for bioassays once rooted and reached 5 cm in height [39].

### Whitefly populations

*Bemisia tabaci* (Gennadius) biotypes B and Q [40] were kindly provided by Dr. Einat Zechori-Fein (Dept. of Entomology, Agricultural Research Organization, Newe Ya'ar Research Center, Israel). The whitefly populations were maintained on cotton plants (*Gossypium hersutum* cv. "Acala") in a growth chamber at 26°C with 60% RH and a 14 h light:10 h dark photoperiod. Adult whiteflies, 3–5 days post emergence were used in all experiments.

### Open field test

The test was conducted in early August 2011 in a commercial field of lemon verbena **(***Lippia citrodora*), a preferred whitefly host. A nearby commercial field of lemon grass (Cymbopogon spp.), which is a non-preferred host, was used as control. Within each field, a 50 m long by 10 m wide plot was selected. The plot was composed of 5 adjacent rows (2 m wide each). Water tubs (10-L) were randomly organized within the plot at a minimum distance of 10 m apart. Two pots (1-L), one with two mature plants (6 month old) of var. '11' and the other with two mature plants of var. '2', were placed together within each water tub. The test was carried out in a randomized complete design with 5 and 4 water tubs (replications) for the lemon verbena and the lemon grass fields, respectively. The colonization of var. '11' and '2' with adult whiteflies was recorded 7 days later and the number of adult whiteflies was counted on the entire plant. One-Way Analysis of Variance (ANOVA) was performed to analyze the open field experiment. Tests for normality and unequal variance were carried out with Shapiro-Wilk and Levenes tests, respectively; and mean separation was executed with Fisher's Protected LSD.

### Choice bioassays

#### Plant-based choice bioassays

A T-shaped glass olfactometer (leg length: 10 cm x arm length: 5 cm with 8 mm internal diameter) was connected to two plastic boxes (10 cm long x 10 cm wide x 4 cm height) that were designated as decision chambers A and B. Each of the decision chambers had two opposite (5 x 5 cm) windows that were sealed with a 50 mesh net (S1a Fig). A male-female mixture of 10 whiteflies were transferred with the aid of a Pasteur pipette, released into the olfactometer leg and were allowed to move to the olfactometer arms to choose between the chambers A or B. The choice bioassay experiments were conducted in the laboratory at 25°C in day light. The system did not include an air stream.

The plant based bioassays were executed with two designs: (i) plant—plant design, in which a single plant of var. '2' and '11' were each placed in the opposite decision chamber; (ii) plant—no plant design, in which var. '2' or '11' were each tested separately with the opposite decision chamber kept empty of a plant. The tests were monitored up to 1h with 5 min. intervals, and the whiteflies captured in each decision chamber or remaining in the olfactometer leg (i.e. 'wandering') were counted and their numbers were recorded. The bioassays were repeated (trial repeat) 5 to 12 times, Initially, bioassays examined the preference of whitefly biotypes (B & Q) to each rosemary variety ('2' or '11') and later used only biotype B.

For statistical analysis, a single whitefly was considered an experimental unit and the number of whiteflies was counted (counts) and recorded into 3 colonization categories: (i) individuals that arrived at decision chamber 'A', (ii) individuals that arrived at decision chamber 'B', and (iii) 'wandering' individuals that stayed in the olfactometer leg and did not choose either decision chamber. Differences among the three colonization categories at each time point were examined with chi-square analysis, followed by single degree of freedom (df = 1) contrasts with likelihood ratio tests to validate differences between colonization of var. '2' and var. '11'.

#### Limonene and β-caryophyllene *in vitro* choice bioassays

The T-shaped olfactometer system described for the plant-based choice bioassay was used here with 600 mL flasks as decision chambers (S1b Fig). Limonene (Cat # 62118; CAS #: 5989-27-5) and β-caryophyllene (Cat #: W225207, CAS #: 87-44-5) were purchased from Sigma-Aldrich, Israel. A series of concentrations of the tested compounds were randomly selected. Dilutions were prepared in 80% Tween 20 solution in distilled water to the final concentrations tested. A solution of 80% Tween 20 in distilled water served as control. The emulsions were dripped onto filter paper discs (3 cm in diameter) and transferred into the decision chambers. The system was laid horizontally and placed against a black background to assist in monitoring the whitefly movement. The whiteflies were released into the olfactometer leg and their preference was monitored up to 1 h with 5 min. intervals. The bioassays were executed with a **compound—no compound design**, in which a tested compound at a designated concentration was placed in decision chamber A and was compared to the control solution that was placed in decision chamber B. The bioassays were carried out 4 times for each compound—concentration combination. Each experimental repeat was performed with 10 individual whiteflies (male-female mix) and the number of whiteflies in each decision chamber was counted. The % preference was calculated and arc-sin transformed. The data were analyzed with one-way ANOVA and means were separated using Tukey-Kramer multiple range test.

### No choice bioassays

#### Cotton plant no-choice bioassay

The essential oils of var. '2' and '11' were diluted (1:1000 v/v) in 80% Tween 20 water emulsion. Cotton plantlets (20 cm long with 5–6 leaves with roots) were dipped in the essential oil emulsion for 10 sec., and immediately placed in a glass tube with tap water and transferred into a plexiglas cage (50 cm long x 30 cm wide x 30 cm height). The cage included two opposite windows (10 cm x 10 cm) sealed with a 50 mesh net. Fifty (n = 50) whiteflies (male-female mixture) were released into each cage and the colonization of the cotton plants was monitored every 24 h up to 120 h. The number of adults inhibiting the plants was counted at each observation. All the experiments were conducted in a growth chamber at 25°C with continuous fluorescent lighting. Cotton plantlets dipped in 80% Tween 20 water solution served as control. The experiments were replicated 3 times, each with 50 (n = 50) whiteflies (male-female mix) per experiment. Linear trend was fitted in Microsoft 'Excel'.

#### Essential oil no-choice bioassay

The essential oils of var. '2' and '11' were distilled in a Clevenger system [41] and kept refrigerated (4°C) until used. The essential oils were diluted (1:1000 v/v) in 80% Tween 20 solution in distilled water. The emulsion (1, 2.5 and 5 μL) was dripped onto filter paper discs (3 cm in diameter) and placed in a 500 mL glass jars to achieve final volatile concentrations of 0.002, 0.005 and 0.01 μL/L (ppm). Tween 20, 80% solution in distilled water served as control. The whiteflies were released into the glass jars and mortality (%) was recorded 1 h later. The experiments were carried out by placing groups of 10 (n = 10) adult whiteflies (male-female mix) each time into the glass jar and were replicated 5 times per each compound-concentration combination. Differences among treatments were examined with chi-square analysis, followed by single degree of freedom (df = 1) contrasts with likelihood ratio tests to validate differences among concentrations.

### Characterization of volatiles

Since different analytical technique have drawback, the full range and complete volatiles' profile in the plant was examined with three different techniques. These were: (i) distillation, (ii) solvent extraction and (iii) Solid Phase Micro-Extraction (SPME). For each technique, five plantlets of the examined varieties '2' or '11' were used.

#### Distillation

Steam distillation of essential oils from plant tissue was performed using a Clevenger system with a 2 L glass canister. 300 g of separated fresh leaves and stems of rosemary from varieties '2' and '11' were distillated in water at 100°C for 1.5 h [41].

#### Solvent extraction

Extraction of volatiles from plant tissue was carried out with 4 g of dry rosemary leaves from varieties '2' and '11' collected from the plant living germplasm collection at Newe Ya'ar Research Center. The vegetative materials were stored in 0.02 L glass sealed vials containing 10-ppm *tert* methyl butyl ether (MTBE) (99.8%) (BIO-LAB, Israel), with an internal standard of isobutylbenzene (IBB) (Aldrich, Israel CAS Number 538-93-2). The extraction ratio was 1 g vegetative material to 0.01 L solvent volume. The samples were shaken for 24 h at room temperature, and then transferred through a Pasteur pipette containing anhydrous sodium sulfate (Merck) and silica gel 60 (230–400 mesh) (Merck) for cleaning, drying and filtering polar components with high molecular weight (e.g. chlorophyll). The cleaned extracted samples were collected in 0.002 L glass bottles, closed with Teflon covers and stored at 4°C before injection into the GC-MS for analysis [41]. GC-MS analysis of the 0.002 L solvent extraction product achieved additional verification of the rosemary components [42].

#### Solid Phase Micro-Extraction (SPME)

A fibrous needle (DVB (Divinylbenzene) /CAR (Carboxen) /PDMS (Polydimethylsiloxane) with medium polarity and 50/30 μm thickness coating (Supelco, PA, USA; cat: 57328 –U) was used for headspace sampling. The needle was inserted into the decision chamber (S1a Fig) and was exposed to the headspace for 15–20 min. Then, the needle was removed from the decision chamber and transferred to the GC-MS for volatile composition analysis [41]. The samples were analyzed using a gas chromatograph / mass spectrometer (Agilent Technologies MSD apparatus, GC- 6890N, MS-5973N- 70ev ionization) equipped with an Rtx-5SilMS fused silica capillary column (Restek) (30 m long, 0.25 μm x id—0.25 mm).

#### GC-MS analysis

The samples were analyzed using a gas chromatograph / mass spectrometer (Agilent Technologies MSD apparatus, GC- 6890N, MS-5973N- 70ev ionization) equipped with an Rtx-5SilMS fused silica capillary column (Restek) (30 m long, 0.25 μm x id—0.25 mm). Following distillation, 30 μl of essential oil were mixed with 0.001 L petroleum ether (40–60°C) (BIO LAB, Israel) and maintained at 4°C in glass bottles until used. Prior to injection into the GCMS, the oil samples were passed through a small column consisting of a Pasteur pipette containing anhydrous Na_2_SO_4_ and salicylic acid (silica gel 60, 230–400 mesh; Merck) to remove water and high molecular weight polar substances that may interfere with the procedure. Helium was used as the carrier gas. The injection port temperature was 250°C and the detector temperatures were 280°C (MS source) and 150°C (MS quad). The column conditions were 50°C for 1 min., 50–200°C at 10 min., 200–280°C at 15 min., and keeping for 10 min. at 280°C. The components were identified by co-injection with standards and by comparison with mass spectra from the computerized libraries Wiley7N and HPCH2205 [41].

#### Statistical analysis

The statistical softwares JMPIN 5.0.1 (SAS Institute, Carry, N.C.) and Sigma Stat./Sigma Plot (version 11.0, System Software Inc.) were used. Analysis of Variance (ANOVA) was conducted in the 'Fit Y by X' platform in JMPIN 5.0.1. Choice and no-choice bioassays were analyzed using tests for proportions that were performed in the 'Distribution' platform in JMPIN 5.0.1 or in the 'Statistic—Rates And Proportion' platform in Sigma Stat. Linear regression analyses and descriptive statistics were calculated in Microsoft 'Excel'. All statistical inferences were carried out at 5% significance level. To limit the bias in the choice and no-choice bioassays the experimental system was disassembled, thoroughly washed and dried following each experiment. All tested whiteflies were discarded and were not reused. A fresh whitefly sample from the maintained cultures was utilized for each experiment. All experiments were repeated in the same location and under similar conditions (light, temperature and relative humidity) in the laboratory or in the growth chamber as indicated.

## Results

### In vivo plant bioassays

**Fig 1:**
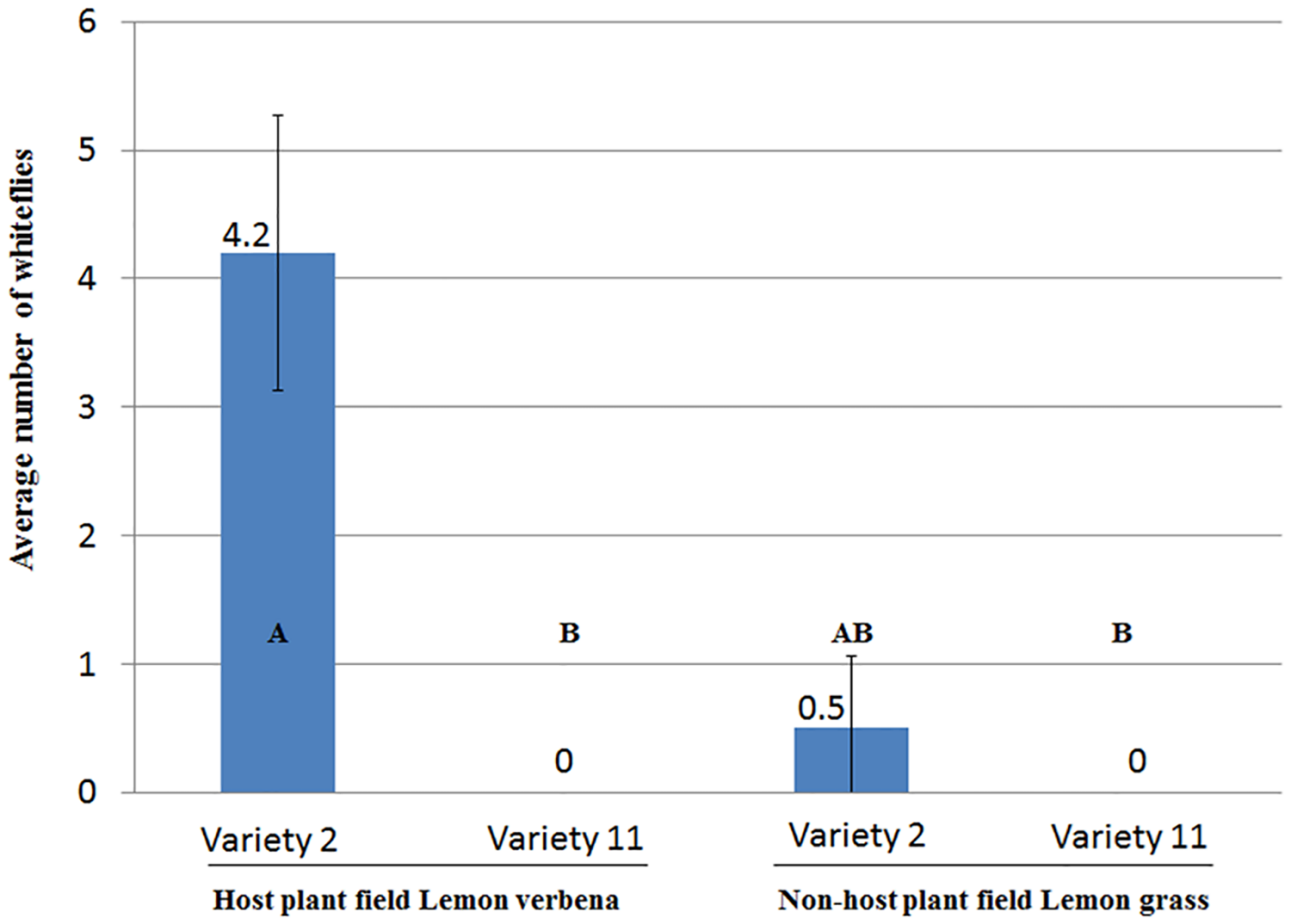
Field colonization of var. 2 and var. 11 plants by whiteflies in commercial fields of Lemon verbena (host field) and Lemon grass (non-host field). Statistical differences among treatments were recorded at *P* = 0.002 following 1-Way ANOVA. Upper-case letters within the histograms indicate differences among means following Fisher's Protected LSD post hoc test (α = 0.05). Error bars represent standard error of the mean (SE ($\bar{X}$)).

The laboratory and field tests examined the interaction between rosemary varieties (var.) '2' and '11' and the local whitefly population. In the field test we expected the allegedly repelling and attracting traits of var. '11' and '2', respectively, to be mitigated by the host lemon verbena and the non-host lemon grass. Nevertheless, the two varieties were populated by whiteflies in the same manner as had been seen previously in the original field observation. Following a 7 day exposure to a severe whitefly infestation in the lemon verbena (host) field, var. '11' was not populated, while var. '2' was significantly more (*P* = 0.002) populated by the insect (Fig 1). At the same time, in the lemon grass field, which is not a host of whiteflies, var. '2' was able to attract whiteflies in a non-supportive surrounding to the insect, yet at a non-significant level (*P*>0.05) compared to var. '11'. The outcome validated that under highly attracting surroundings, var. '2' and '11' attracted and repelled whiteflies, respectively.

**Fig 2:**
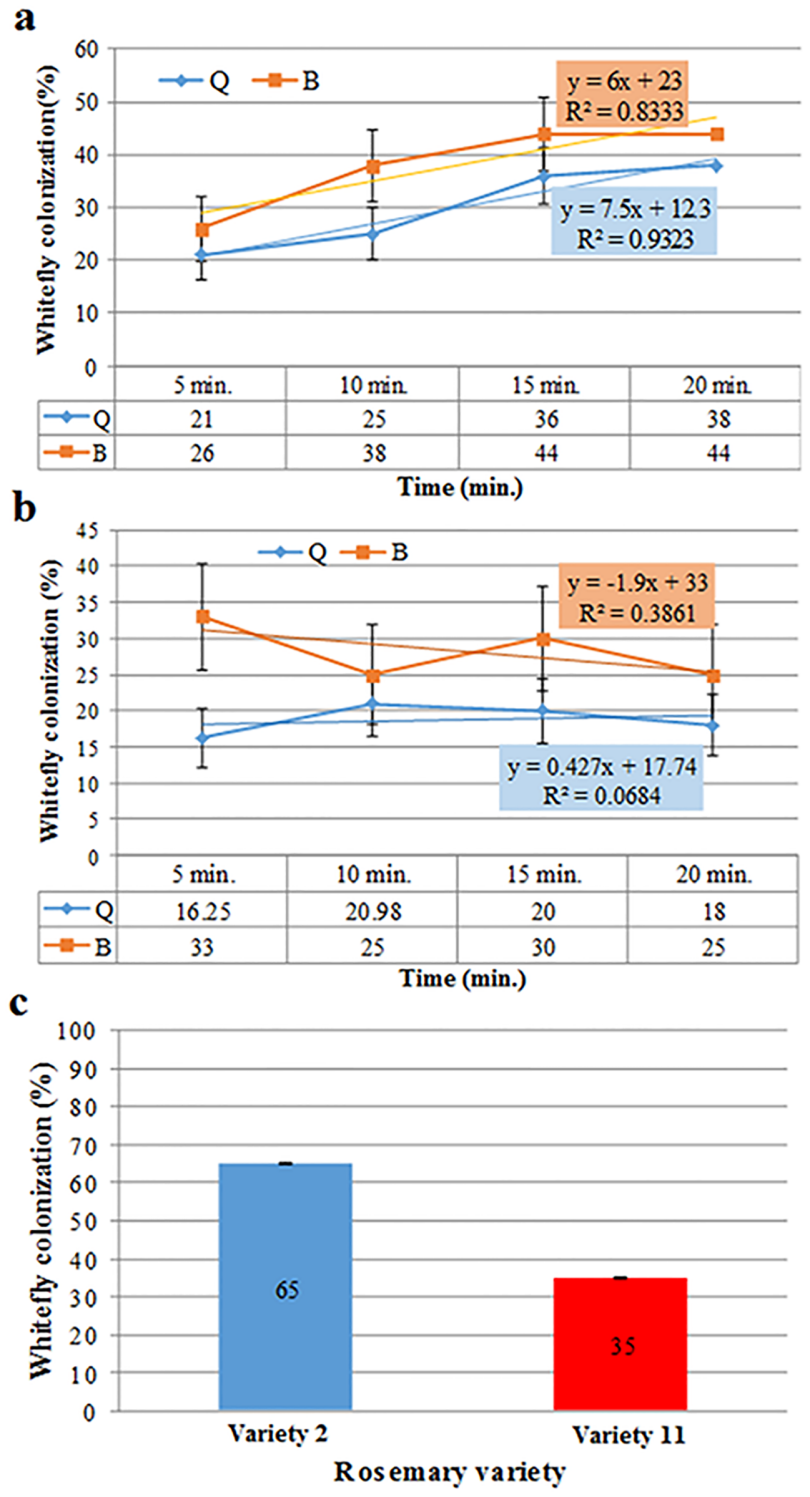
Colonization kinetics of biotypes Q and B on rosemary varieties 2 (A) and 11 (B) and joint preference (C). Chi-square analysis indicated that biotype and variety were independent of each other (*P*>0.05) and that the preference of both biotypes to each rosemary variety was similar. Hence, the data was jointly analyzed indicating that var. 2 was more attractive than 11 (*P* = 0.0091). Error bars represent standard error of the proportion ($SE\left(\hat{p}\right)$).

Prior to initiating controlled laboratory tests, we examined the preference of the whitefly biotypes B and Q for rosemary varieties '2' and '11'. In plant-based choice bioassays it was evident that biotype and variety were independent of each other (*P*>0.05) (Fig 2A & 2B). Therefore, the data was pooled and analyzed together, indicating that both biotypes had greater (*P* = 0.0091) preference for var. '2' than for var. '11' (Fig 2C). Henceforth, all additional experiments were carried out with biotype B, which is also the more common biotype in the region.

**Fig 3:**
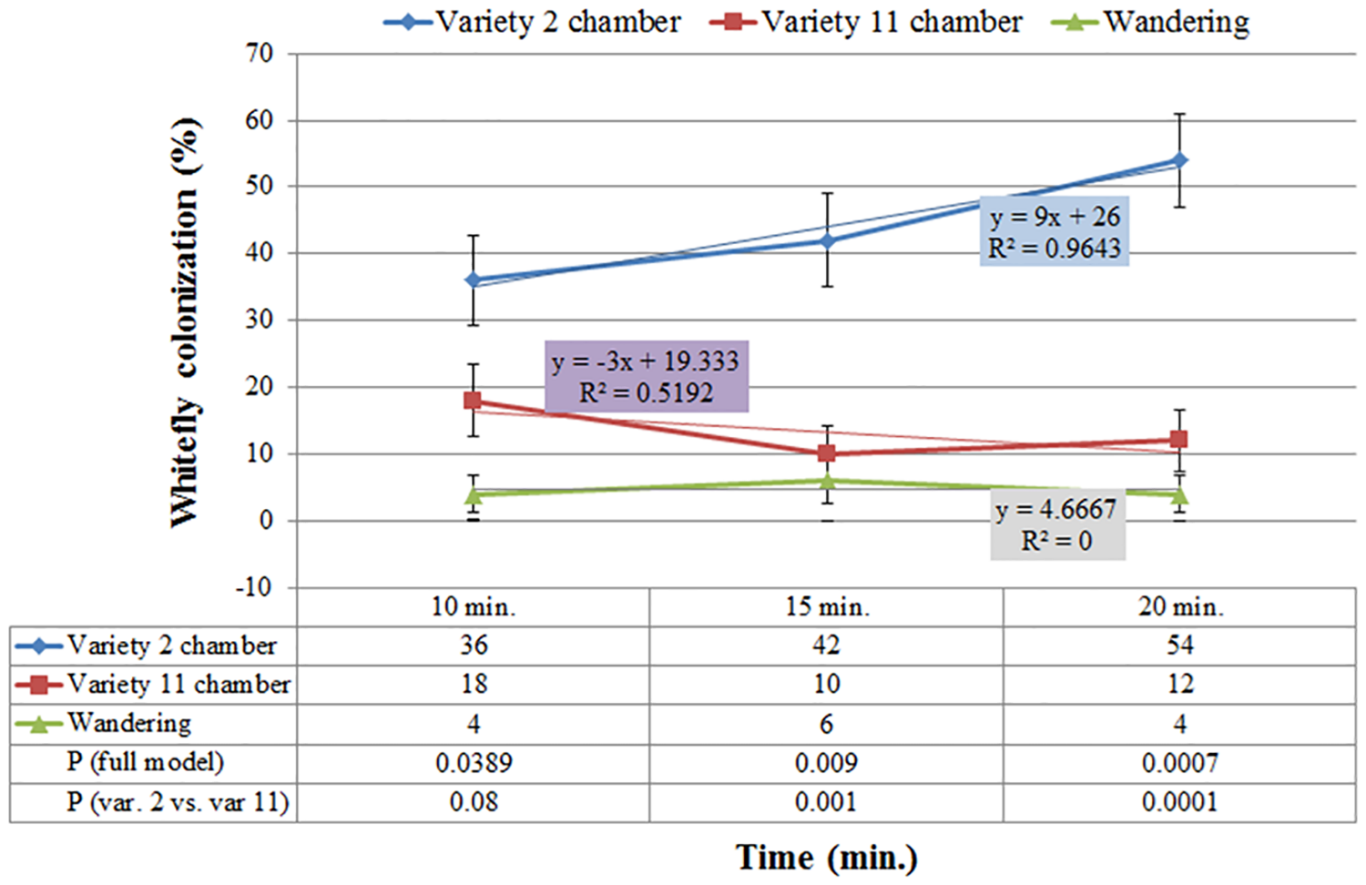
Kinetics of whitefly preference between rosemary vars. 2 and 11. Chi-square analysis identified differences in outcomes among categories (*P*<0.05) throughout the duration of the experiment. Over time, more whiteflies colonized var. 2 chamber than var. 11 with *P* values (var. 2 vs. var. 11) of 0.08, 0.001 and 0.0001, following 10, 15 and 20 minutes of exposure, respectively. Error bars represent standard error of the proportion ($SE\left(\hat{p}\right)$).

**Fig 4:**
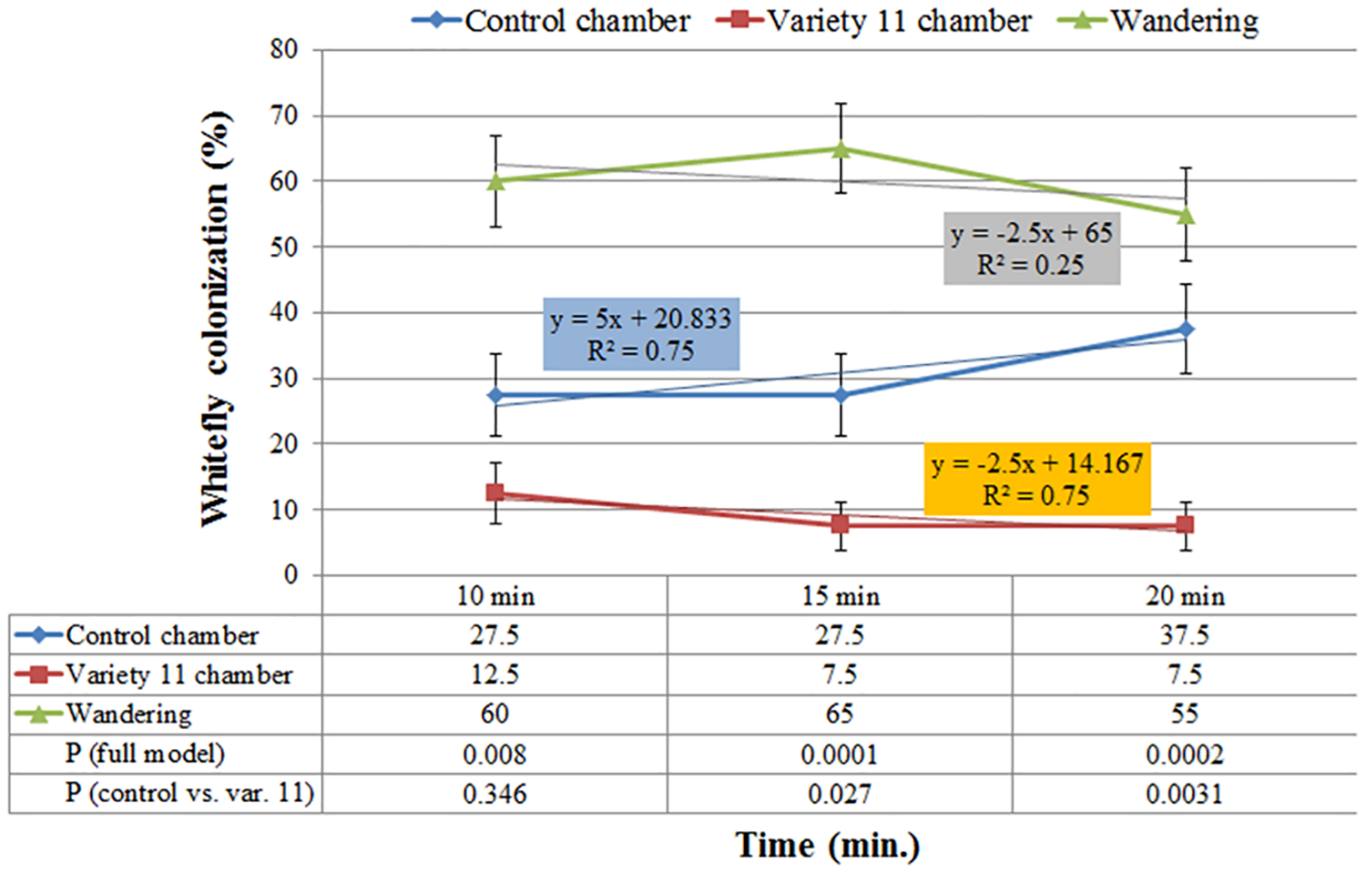
Kinetics of whitefly preference to rosemary var. 11 and unoccupied control chamber. Chi-square analysis identified differences in outcomes among categories (*P*<0.05) throughout the duration of the experiment. Over time, more whiteflies colonized the unoccupied control chamber than var. 11 chamber with *P* values (control vs. var. 11) of 0.027 and 0.0031 following 15 and 20 minutes of exposure, respectively. Error bars represent standard error of the proportion ($SE\left(\hat{p}\right)$).

**Fig 5:**
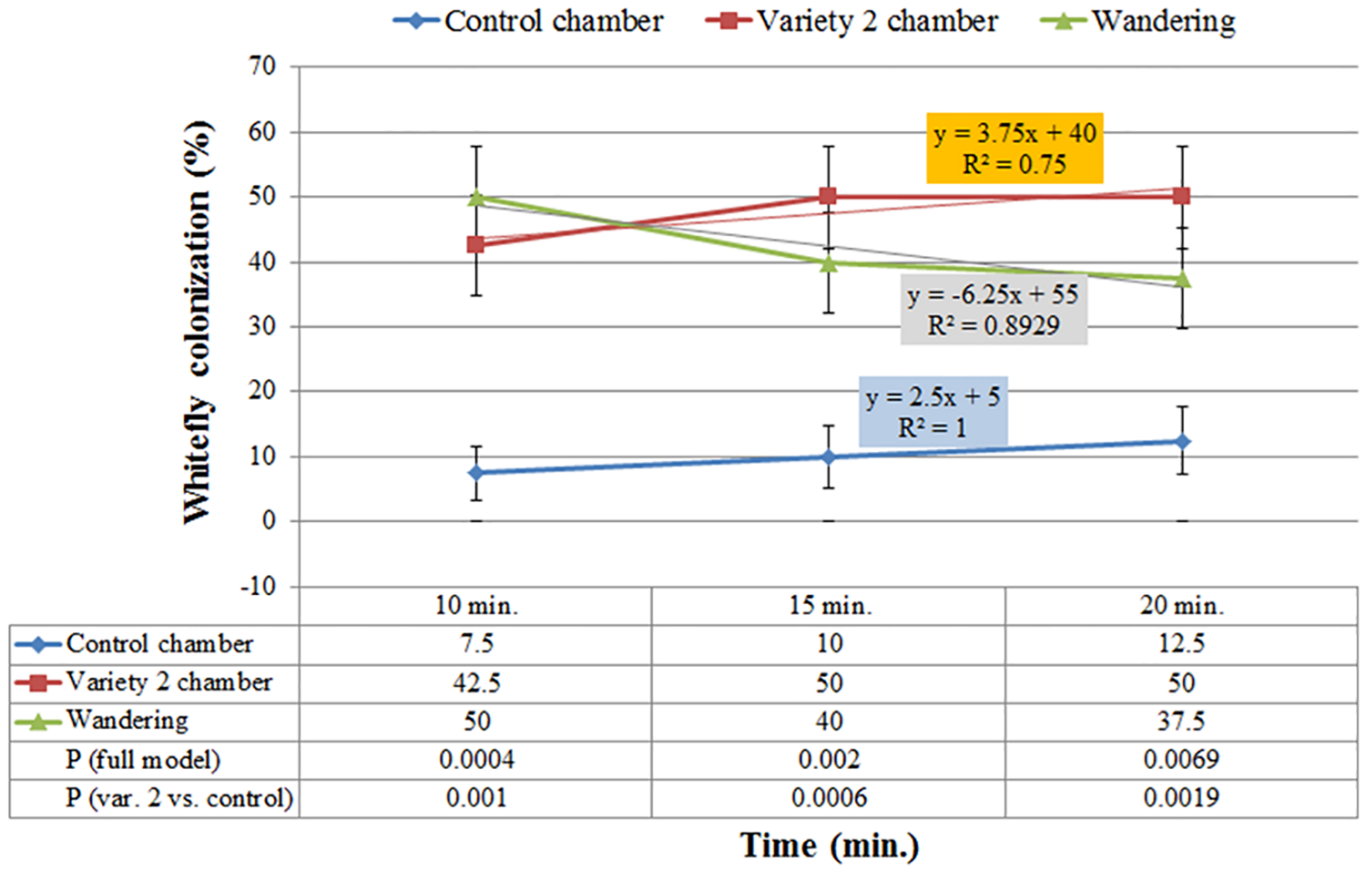
Kinetics of whitefly preference between rosemary var. 2 and the unoccupied control chamber. Chi-square analysis identified differences in outcomes among categories (*P*<0.05) throughout the duration of the experiment. Over time, more whiteflies colonized var. 2 chamber than the unoccupied control chamber with a *P* values (var. 2 vs. control) of 0.001, 0.0006 and 0.0019 following 10, 15 and 20 minutes of exposure, respectively. Error bars represent standard error of the proportion ($SE\left(\hat{p}\right)$).

As colonization of a plant host is a process, it was essential to explore its kinetics. Figs 3, 4 and 5 present the kinetics of whitefly preference between var. '2' and '11'. Statistically significant differences (*P*≤0.05) were observed for colonization categories (variety, control or 'wandering') throughout the duration of the experiment. Over time, more whiteflies occupied the chamber of var. '2' than that of var. '11' with *P* values ('2' vs. '11') of 0.0389, 0.009 and 0.0007, following 10, 15 and 20 minutes of exposure, respectively. Linear trend lines for var. '2' colonization consistently had a positive colonization on time slope (y = 9x+26, R^2^ = 0.9643), unlike the negative colonization on time slope calculated for var. '11' (y = -3x+19.3, R^2^ = 0.5192), asserting a greater attraction to var. '2' than to var. '11' (Fig 3). Comparison of var. '11' to a non-occupied chamber (control) supported its repellency traits. Over time, fewer whiteflies occupied var. '11' chamber than the control chamber with *P* values ('11' vs. control) of 0.027 and 0.003 following 15 and 20 minutes of exposure, respectively. Furthermore, the colonization of the control chamber had a positive colonization on time slope (y = 5x+20.833, R^2^ = 0.75), whereas the colonization of var. '11' had a negative one (y = -2.5x+14.2, R^2^ = 0.75), supporting the observation that var. '11' was repelling the insects over time (Fig 4). On the other hand, var. '2' demonstrated attraction properties by having substantially more whiteflies populating its chamber than the control chamber. A single degree of freedom (df = 1) contrast for var. '2' compared to the control indicated significance at *P* values of 0.001, 0.0006 and 0.0019 following 10, 15 and 20 minutes of exposure, respectively. As expected the colonization of var. '2' had a positive colonization on time slope (y = 3.75x+40, R^2^ = 0.75), which was 1.5-fold greater than that calculated for the control (y = 2.5x+5, R^2^ = 1), pointing out that var. '2' attracted more whiteflies (Fig 5).

**Fig 6:**
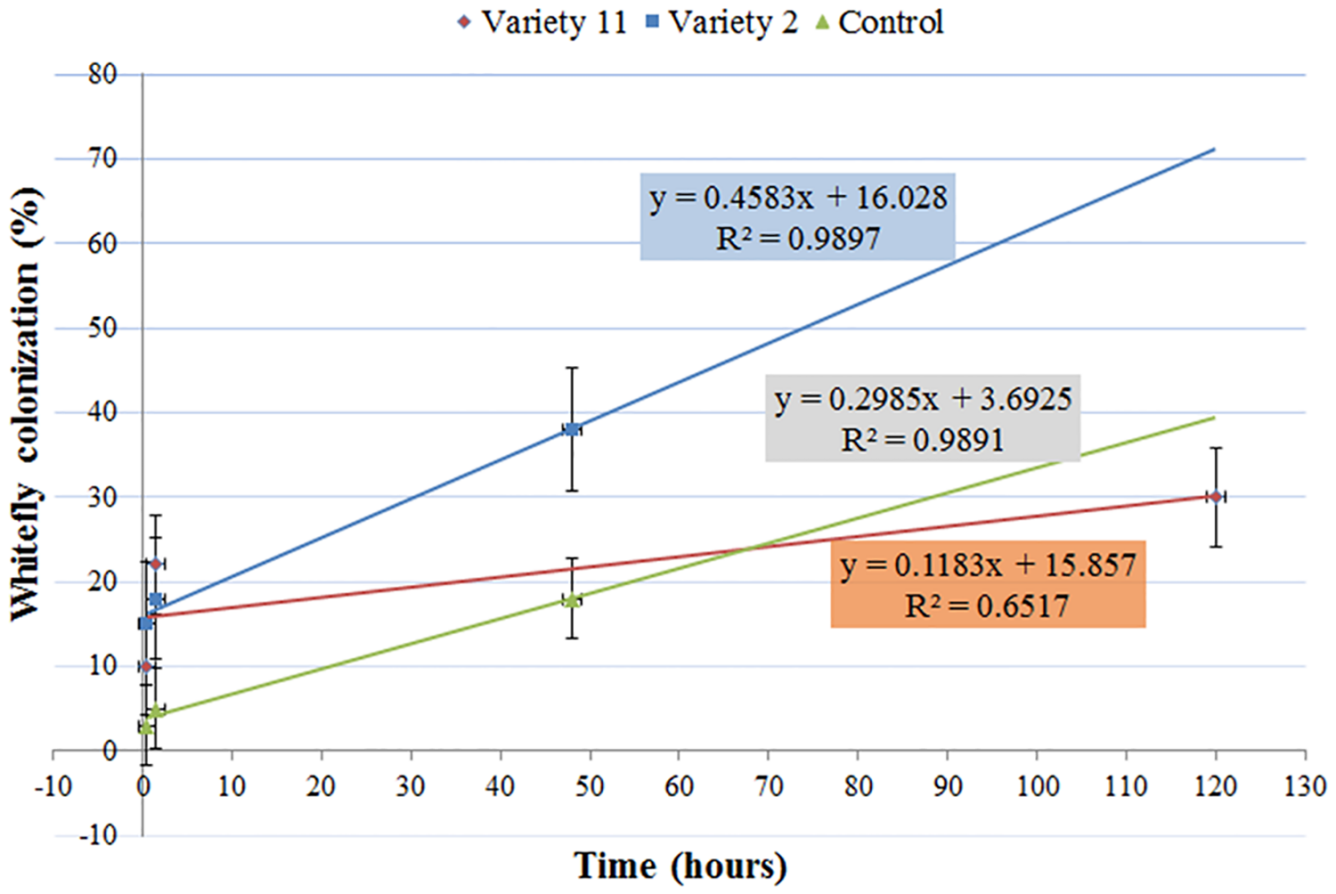
Kinetics of whitefly colonization of cotton plants dipped in the essential oil emulsions of vars. 2 or 11. Control cotton plants were dipped in 80% Tween 20 solution. The colonization of cotton plants dipped in the essential oil emulsion of var. 2 had a 67.4% and 34.8% higher rates than the colonization of cotton plants dipped in the essential oil emulsion of var. 11 or in 80% Tween 20 solution control, respectively. Colonization of the control cotton plants that were dipped in the 80% Tween 20 solution control treatment was 50% higher than the colonization of cotton plants dipped in the essential oil emulsions of var. 11, but not significantly higher (*P*>0.05). Error bars represent standard error of the proportion ($SE\left(\hat{p}\right)$).

Fig 6 presents the kinetics of whitefly colonization of cotton plants dipped in the essential oil emulsions of var. '2', '11' or in 80% Tween 20 solution during no-choice experiments. Cotton is a preferred host for whiteflies and is used to maintain whitefly populations in the lab. Therefore, whitefly attraction to cotton was expected to be mitigated by the repelling properties of var. '11' essential oil, and to be increased by the application of var. '2' essential oil. Cotton plants dipped in the essential oil emulsion of var. '2' had 67.4% and 34.8% higher rates of colonization than cotton plants dipped in the essential oil emulsion of var. '11' or in 80% Tween 20 solution, respectively. The colonization rate of the control cotton plants that was numerically 50% greater than the colonization of cotton plants dipped in the essential oil emulsion of var. '11', however, not significantly (*P*>0.05). These results further supported the attraction of var. '2'. Yet, it seems that the repelling trait of var. '11' was mitigated by the cotton plant at the concentration examined. In all, the outcome of the colonization kinetics examination by choice bioassays indicated that selection of the rosemary varieties by whiteflies was an active process, in which the whitefly responded to volatiles emitted from the plant.

### In vitro bioassays

**Fig 7:**
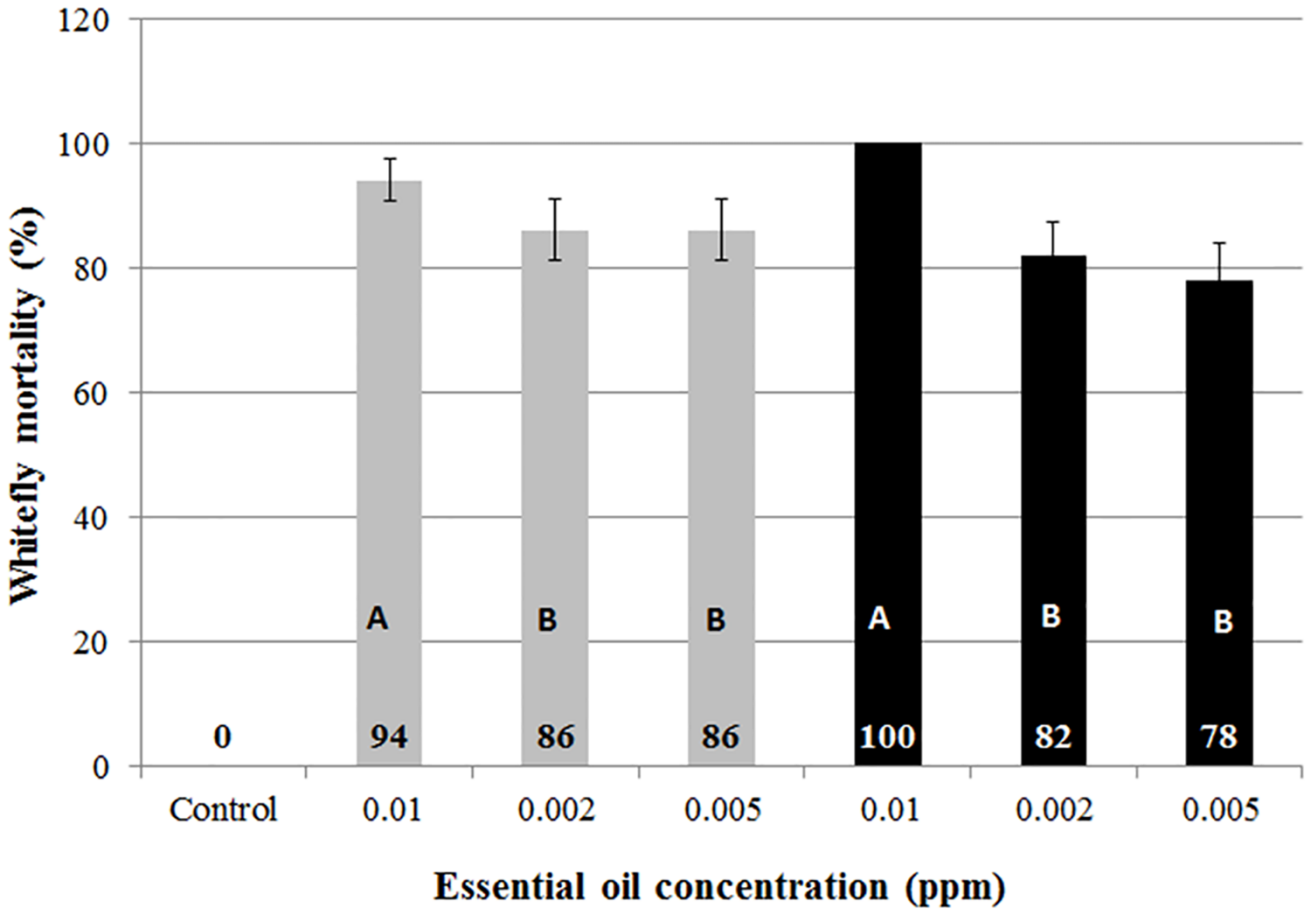
Whitefly mortality (%) following 60 min. of exposure to the essential oil volatiles of var. 2 (black bars) or 11 (grey bars) in 500 mL flasks. Error bars represent standard error of the mean (SE ($\bar{X}$)) and upper-case letters represent statistical differences following 2-way ANOVA and mean separation using Tukey-Kramer multiple range test at α = 0.05.

Plant volatiles are a cue to attraction and repellency. The direct effect of the essential oil in no-choice bioassays demonstrated variability in essential oil lethality and its association with essential oil concentration. Whitefly mortality (%) following 1h of exposure to the essential oil volatiles of var. '2' and '11' in 500 mL flasks ranged between 78–100% and 86–94%, respectively (Fig 7). A variety*concentration and differences between varieties were not recorded. Statistical differences were attained for concentrations, where 0.01 ppm caused greater (*P*<0.001) mortality than 0.002 and 0.005 ppm. (Fig 7). The ecological aftermath of the present case-study is that concentration, rather than volatile composition of the residue emitted to the environment by the non-infested plant affects the active selection by the insect.

**Fig 8:**
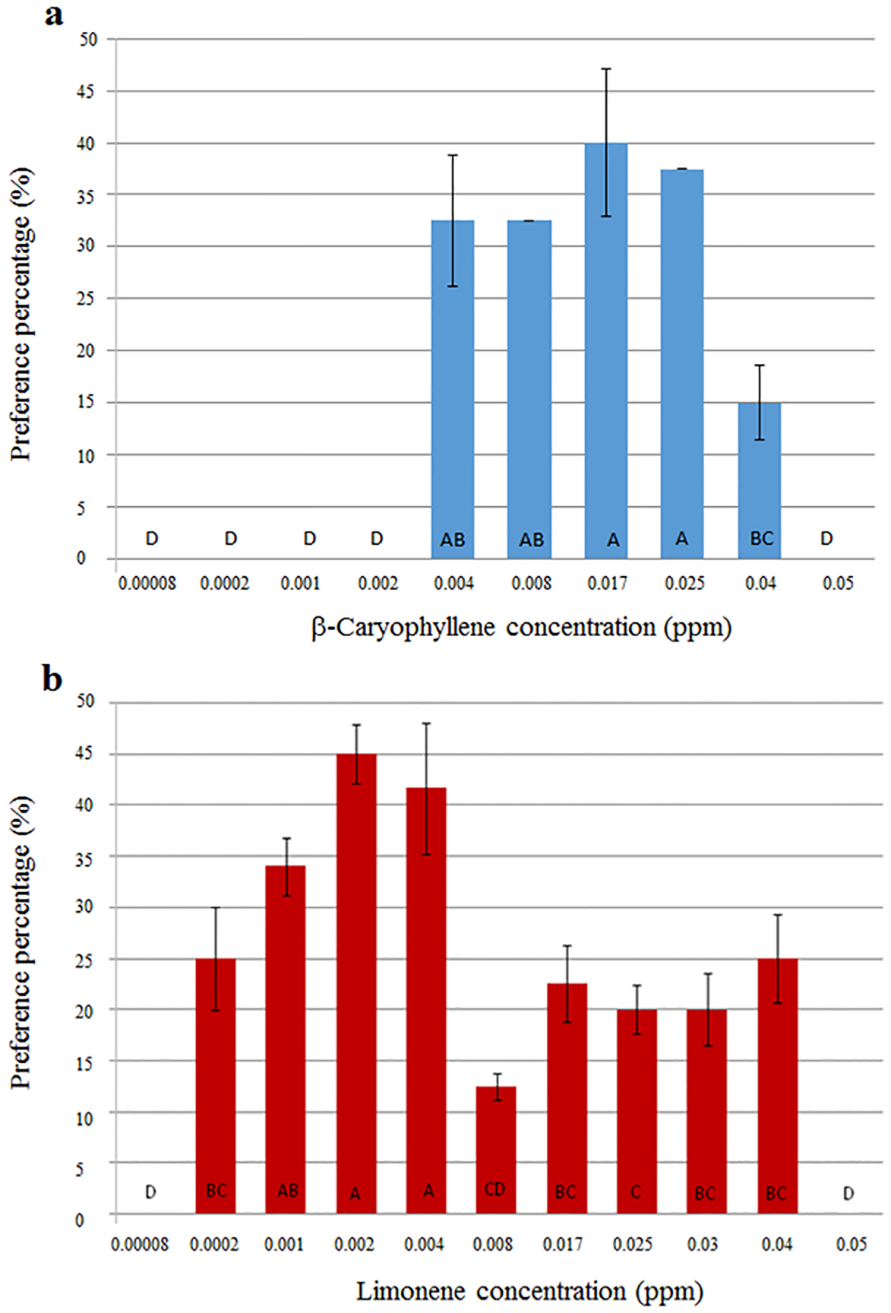
Effect of β-Caryophyllene (A) and Limonene (B) concentrations on whitefly preference *in vitro*. The insects had greater preference to moderate concentrations of the compounds, showing lesser preference to the highest and/or lowest concentrations of *limonene* and β-Caryophyllene, respectively. Furthermore, the similar pattern of attraction was recorded with β-Caryophyllene at concentration 10-fold higher than those experiments of *limonene*. Histograms present % mean preference. Error bars represent standard deviation of the mean. Upper-case letters represent significant statistical differences among concentrations following ANOVA and mean separation using Tukey-Kramer range test at α = 0.05.

The volatile composition of the two varieties was analyzed by three different analytical techniques (distillation, SPME and extraction) and identified compounds that were unique to each variety as well as those that were shared by both of them. Tables 1 and 2 summarize the outcome of the analysis. The compounds α-pinene, camphene, limonene, 1,8-cineole, camphor, borneol, verbenone and bornyl acetate, were shared by both varieties (Table 1). Verbenon and α-pinene were the two major compounds shared. The amounts of camphene, 1,8-cineole and verbenon differed between var. '2' and var. '11' in non-negligible (0.05 < *P* ≤ 0.09) amounts. The amount of camphor differed significantly (*P*<0.001) between the two varieties, with higher concentrations in var. '2' than in var. '11'. Among the unique volatiles identified were β-caryophellene and methyl chavicol, which were distinctive to var. '2' and '11', respectively. Of the recorded compounds, limonene and β-caryophellene were previously reported to be associated with whitefly attraction and repellency to plants [43]. There effects were examined in choice experiments, indicating a preference of *B*. *tabaci* adults for β-caryophyllene at concentrations ranging between 0.004 and 0.04 ppm (Fig 8A & 8B). The highest level of attraction to β-caryophyllene was 40% and was recorded at 0.016 ppm. Increasing the concentration of this volatile up to 0.04 ppm caused a decrease (*P*<0.05) in preference, attracting only 15% of the adult whiteflies. Limonene attracted at concentrations ranging between 0.0002 and 0.008 ppm. The highest level of preference recorded was 45% at a concentration of 0.0002 ppm, while at 0.008 ppm whitefly preference was 32% lower (13%, *P*<0.05). In both cases, the highest concentrations were the least preferred over the moderate ones, and for limonene the lowest concentration was also significantly less (*P*<0.05) attractive for the whitefly. Of note was the 5 to 20-fold difference between the concentrations of the two compounds; yet, both compounds yielded similar attraction levels across concentrations.

**Table 1:** Quantity and summary statistics of major (>1%) volatile constituents identified at both rosemary varieties '2' and '11' following evaluations with three different analytical methods.

Volatile constituent	Variety '11'						Variety '2'						*P*
	Distillation	SPME	Extraction	*Min*.*—Max*.	*Median*	*Mean* ± *stdev*	Distillation	SPME	Extraction	*Min*.*—Max*.	*Median*	*Mean* ± *stdev*	
a-Pinene	16.25	35.43	26.33	16.2–35.4	26.33	26 ± 9.5	21.70	30.39	29.77	21.7–30.3	29.77	27.29 ± 4.85	ns
Camphene	4.07	6.43	7.20	4.0–7.2	6.43	5.9 ± 1.6	8.16	8.92	11.90	8.1–11.9	8.92	9.66 ± 1.97	0.064
Limonene	3.29	7.58	1.85	1.8–7.5	3.29	4.2 ± 2.9	4.15	9.80	1.77	1.7–9.8	4.15	5.24 ± 4.12	ns
1,8-Cineole	6.31	6.73	8.67	6.3–8.6	6.73	7.2 ± 1.2	11.37	10.07	17.67	10.0–17.6	11.37	13.04 ± 4.06	0.078
Camphor	2.86	1.02	1.92	1.0–2.8	1.92	1.9 ± 0.9	10.26	7.80	9.15	7.8–10.2	9.15	9.07 ± 1.23	0.001
Borneol	4.07	1.12	3.94	1.1–4.0	3.94	3.0 ± 1.6	5.13	2.00	6.52	2.0–6.5	5.13	4.55 ± 2.32	ns
Verbenone	18.35	4.68	25.68	4.6–25.6	18.35	16.2 ± 10	2.13	0.38	2.61	0.3–2.6	2.13	1.71 ± 1.17	0.079
Bornyl acetate	8.91	8.41	7.15	7.1–8.9	8.41	8.1 ± 0.9	9.02	4.20	2.85	2.8–9.0	4.20	5.36 ± 3.24	ns

Quantity (%) of volatile constituent according to each analytical method. Minimum-maximum, median and mean ± standard deviation represent the consolidated level (%) of each volatile constituent of the three analytical methods (distillation, extraction and SPME). *P* represents, within a row, the differences between the mean values of each constituent in varieties '11' and '2' following a single degree of freedom (df = 1) contrast(α = 0.05). *P* values ≤ 0.05 indicate significant differences; 0.05 < *P* ≤ 0.09 indicate a non-negligible difference; and 'ns' indicates lack of differences (*P* > 0.09).

**Table 2:** Summary statistics of volatile constituents unique to each rosemary variety following evaluations using three different analytical methods.

Variety	Volatile constituent	Oil	SPME	Extract	Min.–Max.	Median	Mean ± stdev
'11'	d-3-Carene	0.02	0.03	0.00	0.00–0.03	0.02	0.02 ± 0.01
	ortho-Cresol methyl ether	0.00	0.07	0.00	0.00–0.07	0.00	0.02 ± 0.04
	Filifolone	0.00	0.31	0.00	0.00–0.31	0.00	0.10 ± 0.18
	trans-Pinocarveol	0.64	0.16	0.47	0.16–0.64	0.47	0.43 ± 0.24
	trans-Pinocamphone	0.11	0.07	0.11	0.07–0.11	0.11	0.10 ± 0.02
	cis-Pinocamphone	0.64	0.38	0.00	0.00–0.64	0.38	0.34 ± 0.32
	Methyl chavicol	0.00	1.56	0.00	0.00–1.56	0.00	0.52 ± 0.90
	Thymol methyl ether	0.11	0.08	0.00	0.00–0.11	0.08	0.07 ± 0.06
	Carvone	0.26	0.09	0.00	0.00–0.26	0.09	0.11 ± 0.13
	Isopiperitenone	0.71	0.19	0.00	0.00–0.71	0.19	0.30 ± 0.37
	trans-Pinocarvyl acetate	0.42	0.13	0.00	0.00–0.42	0.13	0.18 ± 0.21
	Myrtenyl acetate	0.54	0.13	0.23	0.13–0.54	0.23	0.30 ± 0.22
	Methyl eugenol	1.18	0.12	0.48	0.12–1.18	0.48	0.59 ± 0.54
'2'	β-Caryophyllene	0.96	0.65	2.08	0.65–2.08	1.23	1.23 ± 0.75
	3-Octanol	0.14	0.16	0.00	0.58–0.76	0.70	0.68 ± 0.09
	a-Ylangene	1.27	2.58	0.83	0.18–0.35	0.19	0.24 ± 0.09
	6,9-Guaiadiene	0.76	0.58	0.70	0.03–0.11	0.06	0.06 ± 0.05
	a-Humulene	0.06	0.03	0.11	0.25–0.84	0.57	0.55 ± 0.30
	a-Amorphene	0.13	0.04	0.20	0.04–0.20	0.13	0.12 ± 0.08
	d-Amorphene	0.57	0.25	0.84	0.00–0.21	0.04	0.08 ± 0.11

All values are % units, where minimum—maximum, median and mean ± standard deviation represent the consolidated quantity of the volatile constituent recorded by distillation, extraction and SPME analyses of the plant tissue.

## Discussion

Volatile cues have been the subject of intensive research over the last decade. Such research was aimed at the identification of specific volatiles that have attracting or repelling effects on insects [44]. Plant volatile blends consist of major semiochemicals such as terpenes and other green leaf volatiles, but also minor compounds that can have diverse effects of attracting or repelling insects [44]. These volatiles or blends of semiochemicals may facilitate the recognition of plant hosts by insects from a distance. Moreover, these volatiles may also be emitted from plant hosts that belong to closely related species, depending on the insect's olfactory system and how well the insect perceives those signals from host plants [45]. As mentioned, *B*. *tabaci* is an acutely polyphagous insect and feeds on hundreds of plant species [1,2,3,4,5,6,8]. This makes it difficult to determine whether this insect recognized plant hosts based only on the sense of smell. Moreover, the recognition of specific volatiles or their preference probably involves visual cues as well. The diversity of plant hosts on which *B*. *tabaci* feeds suggests that the insects may be familiar with a wide diversity of plant volatiles and that there may be only minor differences among the host plants. Previous studies have attempted to identify plant volatiles from various plants that attract or repel *B*. *tabaci*. Gas chromatography was used to identify such volatiles from tomato, tobacco, cabbage, cotton, cucumber and celery [43]. Five of the tested plant hosts showed high levels of attractiveness, while only celery showed repellency. Chemical analysis showed that fatty acids, terpene compounds, aromatic compounds and short-chain or middle-length -chain hydrocarbons were the major chemicals identified in these plants [43]. The essential compounds of the volatile fraction were 2-hexenal and 3-hexen-1-ol. Terpenoids were the most abundant chemical compounds among the host plants and included α-pinene, β-myrcene, ocimene, limonene, β-phellandrene and β-caryophyllene. Consequently, the different host plants varied in their relative contents of various plant oils. For example, one of the chemicals, limonene, was a principal ingredient of the volatile component of celery while it appeared at very low concentrations in the other plants selected. Thereupon, while all the compounds were thought to attract *B*. *tabaci*, the presence of limonene at a very high concentration in celery suggested that it has a repelling action [43].

In our study, the essential oil analysis of rosemary varieties '2' and '11' indicated the presence of most of the above-mentioned chemical compounds (Tables 1 and 2). Most notably, β-caryophyllene was found in the attracting var. '2', while limonene was observed in both varieties, including the repelling var. '11' (Tables 1 and 2). This outcome may suggest that β-caryophyllene and limonene have a general effect on *B*. *tabaci* in different host plants. Both limonene and β-caryophyllene were tested in choice assays in the lab, and the results showed that the preference levels of *B*. *tabaci* of each compound were associated with the compounds' concentration (Fig 8). At higher concentrations, both compounds had repelling effects. These results and the results obtained in the whole plant examinations and assays described for other plant hosts suggest that these two compounds do not act as single volatiles. More likely, they act in blends with other volatiles, leading to different behavior among *B*. *tabaci* adults.

Additional studies have also shown that terpenoids were major compounds that played an important role in *B*. *tabaci-*tomato interactions. In tomato, the sesquiterpenes zingiberene and curcumene, and the monoterpenes β-cymene, α-terpinene and α-phellandrene had the strongest effects in choice bioassays [46]. These terpenes also elicited a response of receptors on the insects' antennae. Some monoterpenes such as β-myrcene showed no activity in both examinations [47]. Some of these compounds, which were identified in the present study (Tables 1 and 2), were also identified in tomato, suggesting that the response of *B*. *tabaci* is specific across different plant hosts [43,46]. Interestingly, when tomato plants were infested with another sap-sucking generalist, the green peach aphid Myzus persicae [47], volatiles produced following the infestation repelled *B*. *tabaci*, while attracting six natural enemies of the aphid. Furthermore, an increase in aphid densities on the leaves directly increased the repellency to *B*. *tabaci* [47]. Unfortunately, the volatiles emitted following the aphid's infestation were not analyzed chemically. Yet, similarities between the volatiles emitted by the aphid and the volatiles emitted by the whitefly are likely to be found.

In summary, this study demonstrated that volatiles emitted from two different rosemary varieties, each a unique chemotype, played an essential role in *B*. *tabaci* preference for these plants. β-caryophyllene, a widespread sesquiterpene, was found to be a unique constituent in the essential oil of var. '2' in all analyses (SPME, solvent extraction and distillation). This compound is known to take part in plant defense. However, the role of β-caryophyllene as attracting *B*. *tabaci* to rosemary plants is new and innovative information, which is contrary to literature reports that mentioned its role in the defense mechanism of non-floral tissues [48]. The range of β-caryophyllene concentrations, which attract or repel the insect, was wider than the range recorded for limonene, which was present in both varieties. Thus, *B*. *tabaci* responded differently to various concentrations of volatile compounds, suggesting that the insect identified the attractiveness of the host by sensing the type and concentration of volatiles emitted from it. The results of this study are a significant step in deciphering the cues regulating whiteflies' attraction to their host. Utilization of these results would assist in developing eco-friendly methods of managing *B*. *tabaci* infestations via manipulation of these cues.

## Supporting information

S1 Fig The experimental setup used for choice and no-choice tests. Two cube-shaped plastic chambers containing rosemary young plants for comparing the preference of adult whiteflies were connected with a T-shaped glass tunnel on a dark background (a). No-choice laboratory experiments for a specific volatiles were conducted using the T- shaped apparatus for connecting two horizontally laid bottles (0.6 L each), and placed on a black background (b). The insects were introduced into the glass tunnel and allowed to choose between the bottles, led only by their sense of smell.
(PDF)
