# Supplementary material for: Whitefly attraction to rosemary (Rosmarinus officinialis L.) is associated with volatile composition and quantity
Source: PLoS One. 2017 May 12;12(5):e0177483. doi: 10.1371/journal.pone.0177483 (PMC5428955; doi:10.1371/journal.pone.0177483)
Supplement: S1 Fig — Two cube-shaped plastic chambers containing rosemary young plants for comparing the preference of adult whiteflies were connected with a T-shaped glass tunnel on a dark background (a). No-choice laboratory experiments for a specific volatiles were conducted using the T- shaped apparatus for connecting two horizontally laid bottles (0.6 L each), and placed on a black background (b). The insects were introduced into the glass tunnel and allowed to choose between the bottles, led only by their sense of smell. (PDF) [file pone.0177483.s001.pdf]

a

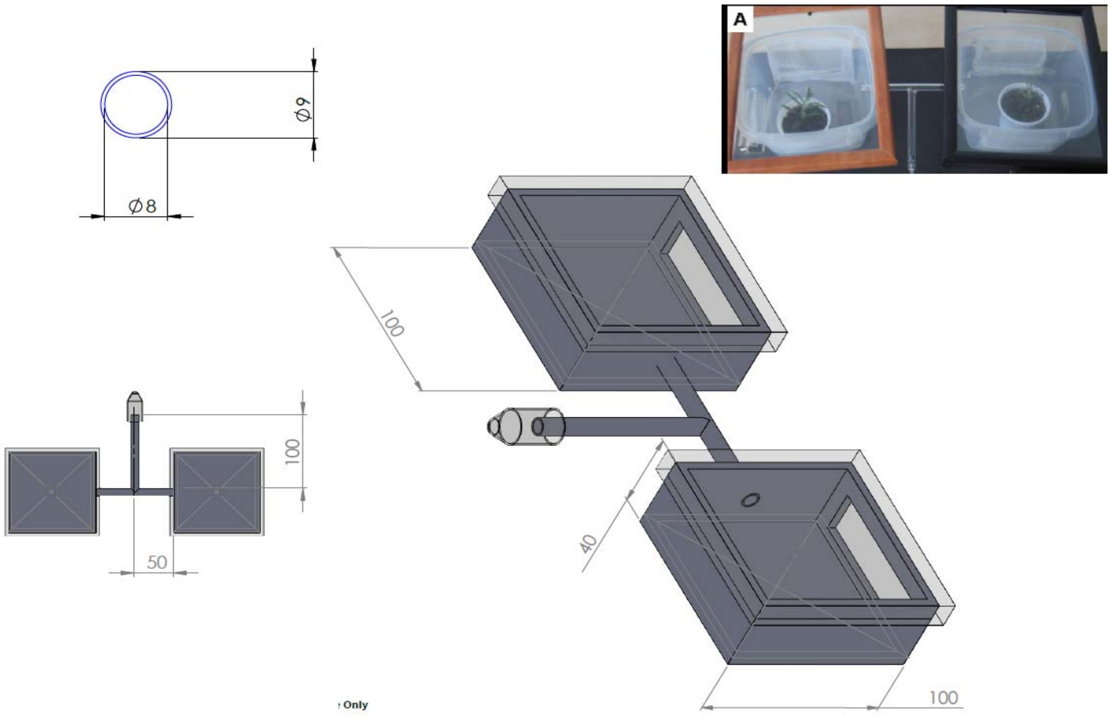

b

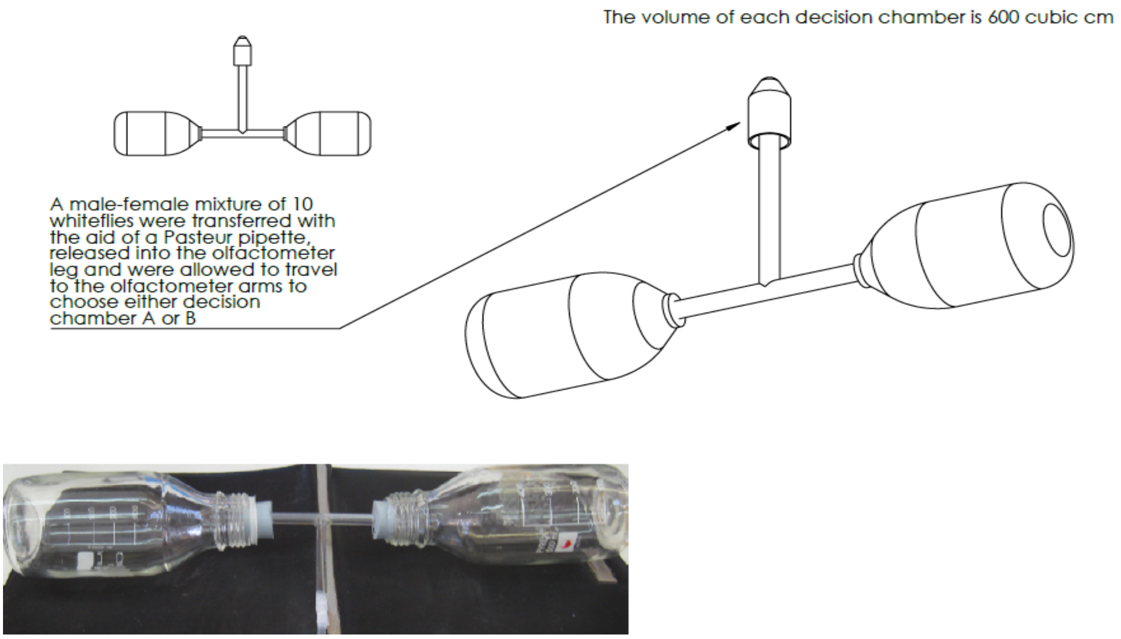

Fig S1. The experimental setup used for choice and no-choice tests. Two cube-shaped plastic chambers containing rosemary young plants for comparing the preference of adult whiteflies were connected with a T-shaped glass tunnel on a dark background (a). No-choice laboratory experiments for a specific volatiles were conducted using the T- shaped apparatus for connecting two horizontally laid bottles (0.6 L each), and placed on a black background (b). The insects were introduced into the glass tunnel and allowed to choose between the bottles, led only by their sense of smell.
